# When and How Does the Job Insecurity of Salespersons Become a Sleep Problem? The Moderating Roles of Organizational Control Systems

**DOI:** 10.3390/healthcare8040422

**Published:** 2020-10-22

**Authors:** Chang Mo Jung, Tae-Won Moon, Won-Moo Hur

**Affiliations:** 1 School of Business Administration, Yonsei University 50, Yonsei-ro, Seodaemun-gu, Seoul 03722, Korea; 2011313041@yonsei.ac.kr; 2 School of Business Administration, Hongik University, 72-1 Sangsu-dong, Mapo-gu, Seoul 121-791, Korea; twmoon@hongik.ac.kr; 3 College of Business Administration, Inha University, 100, Inha-ro, Michuhol-gu, Incheon 22212, Korea

**Keywords:** job insecurity, emotional exhaustion, sleep, formal organizational control system, outcome-based control, behavior-based control

## Abstract

The present study examines the effect of the emotional exhaustion associated with salespersons’ job insecurity on their sleep (i.e., insomnia symptoms). We identified two types of formal organizational control systems (i.e., outcome-based and behavior-based controls) as boundary conditions that strengthen/weaken the positive relationship between job insecurity and emotional exhaustion. To test this moderating effect, we collected online panel surveys from 187 Korean salespersons at two time points, which were separated by three months. Like our predictions, the positive relationship between job insecurity and negative sleep quality (i.e., insomnia symptoms) was found to be mediated by emotional exhaustion. We further found a significant three-way interaction between job insecurity, outcome-based control, and behavior-based control, which is mediated by emotional exhaustion, indicating that the positive relationship between job insecurity and emotional exhaustion was strongest when the outcome-based control and behavior-based control of salespersons were high and low, respectively. The indirect effect of the emotional exhaustion associated with job insecurity on sleep quality was also weakest when the outcome-based control and behavior-based control were both high. These results provide theoretical and practical implications for managing employees in job insecurity contexts.

## 1. Introduction

The economic recession that has taken place over the last few decades and the concomitant increase in uncertainty in the labor market have led employees to feel job insecurity [1]. Job insecurity is referred to as “the uncertainty a person feels in relation to his or her job continuity” [2]. Job insecurity is challenging and poses a critical problem in several industries [3], leading to considerable costs [4]. Previous studies have examined employees experiencing job insecurity across a wide range of occupations and industries [5] and have proposed a model of job insecurity relevant to each job [6]. While job insecurity is a critical issue for service firms [7,8,9], few studies have examined the job insecurity of service employees and, in particular, salespersons. In response to this lack in the literature, we attempt to develop a model of job insecurity that is specifically targeted at salespersons.

Given that job security is a pivotal factor affecting salespersons’ outcomes, it is both theoretically and practically crucial to investigate job insecurity in the sales sector. There are three reasons for this. First, salespersons tend to engage in emotional labor through direct interactions with customers, leading to a high level of emotional exhaustion and job stress [10,11,12]. Salespersons also experience threats of temporary employment and minimum wages [13], which jeopardize their job security [14]. Second, since internet-based sales channels can make salespersons’ roles in an organization obsolete, this makes them especially vulnerable, thus increasing their job insecurity [15]. Third, a salesperson’s job insecurity is considered as a largely unexamined job stressor, which might have important implications for the welfare and performance of salespersons [3]. Thus, it is vital to examine salespersons’ job insecurity and its effects [3,4].

To meet this demand, our research examines how salespersons’ job insecurity impacts on their sleep (i.e., insomnia symptom). This research is based on affective events theory (AET; [16]), which suggests that negative work events trigger negative emotional responses, thus depleting the resources necessary for psychological well-being and deteriorating sleep quality. Deteriorated sleep quality is defined as “difficulty in falling asleep and staying asleep, waking up several times during the night, and not feeling rested upon waking” ([17], p. 683). In this respect, we propose emotional exhaustion (i.e., a negative emotional reaction) as a critical intermediary mechanism linking job insecurity and insomnia symptoms.

Another aim of our research is to investigate the role of organizational control systems in causing the negative effect of job insecurity on salespersons’ sleep. A control system is especially important in explaining the extent to which salespersons are left exhausted when experiencing uncertainty in their job continuity, something which has not yet been sufficiently addressed in the literature. Since organizational control systems are management strategies that are used to manage and monitor employee task performance [18], we expect these two control systems (e.g., outcome-based and behavior-based controls) to differentially influence the effect of job insecurity on emotional exhaustion. The prior research in this area has an important limitation, i.e., the effects of organizational control systems on salespersons are examined separately, which may lead to uncertain or even misleading findings [19,20]. A few studies have investigated the effects of hybrid organizational control systems on salespersons [21,22,23], but the question of how they influence salespersons’ emotional exhaustion remains unanswered.

**Figure 1:**
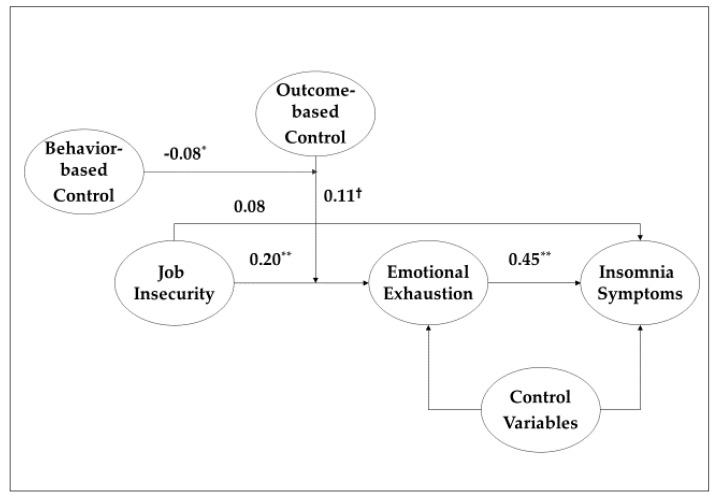
Moderated mediation model. Notes: ^†^ *p* < 0.1, * *p* < 0.05, ** *p* < 0.01. Coefficient: unstandardized coefficient. For parsimony, the control variables (i.e., gender, age, education, job tenure, job status, social desirability bias, and PANAS) are not included in this figure.

To fill this gap, based on the job demands-resources (JD-R) model, which indicates that job-relevant contextual factors, such as job demands and resources, predominantly influence employee outcomes [24], we examine the jointly moderated mediation effects of organizational control systems (e.g., outcome-based and behavior-based controls) on this relationship. That is, we test the jointly moderating effects of organizational control systems on the relationship between salespersons’ job insecurity and sleep, as mediated by emotional exhaustion. The proposed model is illustrated in Figure 1 and is explained in detail in the following sections.

## 2. Theoretical Background and Hypothesis Development

### 2.1. Mediation of Emotional Exhaustion on the Job Insecurity–Sleep Problem Relationship

Job insecurity research has identified stress-related mechanisms as mediators between job insecurity and psychological well-being. Job insecurity, as a major job stressor, has been found to exert a negative effect on psychological well-being by increasing psychological strains, such as anxiety and emotional exhaustion (e.g., [8,25]). While most of the previous studies have focused on psychological well-being and job performance as outcomes of service employees’ job insecurity, a few studies have paid attention to sleep problems or poor sleep quality as influencing factors of job insecurity (e.g., [26,27]). Thus, we investigate the link between job insecurity and sleep problems, as recent studies are increasingly examining sleep as a crucial factor of employee well-being and job performance (i.e., decision making and safety; [28,29]).

Organizational sleep research has conceptualized sleep in terms of the quantity and quality of sleep [17,30,31,32]. Sleep quantity is defined as the amount of time a person remains in a sleeping state [28], while sleep quality is defined as “the ability of an individual to fall asleep and stay asleep, the number of times he/she wakes up at night, and the feeling of restfulness upon waking” ([17], p. 683). Some studies have demonstrated that both sleep quality and quantity have the same effects on outcomes, such as health, well-being, and cognition [28], while others have shown that sleep quality is more strongly correlated with such outcomes than sleep quantity [17,33]. Accordingly, we focus on sleep quality and examine insomnia symptoms, including difficulty falling asleep, difficulty staying asleep, and experiencing nonrestorative sleep [34] as indicators of a poor sleep quality.

While it is possible that a poor sleep quality endangers employees’ job insecurity threats by increasing their emotional exhaustion, we propose that the reverse relationship between job insecurity and sleep quality that is mediated by emotional exhaustion has a stronger theoretical foundation. The positive relationship between job insecurity and emotional exhaustion has been well supported in the literature [25]. Shin and Hur [8] found that the positive link between job insecurity and job performance was mediated by emotional exhaustion. Our proposed mediating effect of emotional exhaustion between job insecurity and insomnia symptoms can be explained by AET, postulating that negative workplace events cause employees to experience negative emotions, which then trigger negative behavioral responses [16]. The job insecurity literature has shown that job insecurity is a primary negative event associated with work. Salespersons experience threats of job insecurity at work, which develops into negative emotional reactions, such as a low level of emotional energy and vigor, as well as a high level of anxiety, helplessness, and hopelessness [35]. We propose that emotional exhaustion triggered by job insecurity leads to negative behavioral reactions, which discourage salespersons from engaging in their work and then negatively affect their sleep quality. Drawing on AET and empirical findings, we propose that job insecurity is positively related to insomnia symptoms by stifling emotional exhaustion, which leads to the following mediation effect:

**Hypothesis** **1.** The positive relationship between salespersons’ experienced job insecurity and insomnia symptoms is mediated by emotional exhaustion.

### 2.2. The Moderating Effect of Organizational Control

Organizational control systems play a key role in determining the contextual factors that influence salespersons’ attitudes and behaviors in a desirable manner [36]. Salespersons often work under an organizational control system (e.g., either outcome-based control or behavior-based control) during service encounters with customers. Experiencing job insecurity in a different context may lead to a different level of emotional exhaustion, unlike that associated with job insecurity by itself. In accordance with the JD-R model, we predict that organizational outcome-based control and behavior-based control have different moderating effects on the link between job insecurity and emotional exhaustion. More specifically, we propose that organizational outcome-based control functions as a job demand, which strengthens the positive association between job insecurity and emotional exhaustion, whereas behavior-based control serves as a job resource, which attenuates this relationship.

A behavior-based control system requires managers to have more time to communicate with salespersons through close monitoring and supervision in order to attain desired goals [37]. Under a behavior-based control system, managers are likely to have more information about salespersons’ effort during service encounters with customers and be able to provide substantial support, care, and managerial direction, which enhances salespersons’ trust in the organization and satisfaction towards their jobs [38,39]. Previous studies have found that a behavior-based control system is positively associated with several employee outcomes, such as sales skills and motivation, job satisfaction, trust in supervisors, and behavior performance [40]. By contrast, outcome-based control systems are based on objective measures of the outcomes (i.e., a salesperson’s sales volume, revenue, or quota attainment) that are demanded by the firm, and salespersons are fully responsible for their accomplishment, with little monitoring of salespersons by management [41]. Under an outcome-based control system, salespersons tend to have substantial autonomy in performing their jobs (e.g., they may choose their own sales strategies) and are tightly evaluated and rewarded for positive outcomes, such as a high sales volume, market share, and profit contribution [38]. Since salespersons’ compensation is determined by output standards, managers provide little support and have little concern for employees, which may lead to a substantial performance risk, job pressure, and role stress on salespersons [40].

We propose that an interaction effect between job insecurity, high outcome-based control, and low behavior-based control may increase emotional exhaustion. Salespersons are likely to take a greater amount of time to fulfill their sales order in the context of a high level of job uncertainty due to the feeling of job insecurity. Based on the JD-R model, a lack of supervisor support and job pressure under an outcome-based control system function as important job demands, which can cause salespersons to be frustrated and discouraged, leading to a decrease in job satisfaction and commitment to organizations [41,42]. Moreover, salespersons are less likely to have perceptions of fairness in terms of rewards and punishments if mangers have less chance to observe and accurately measure certain aspects of salespersons’ contributions [41]. Outcome-based control allows a salesperson to receive compensation based on the extent to which the organizational goals, such as sales volumes, are accomplished, as opposed to the process requirements, which increases job stress and pressure and, in turn, may increase emotional exhaustion. In short, salespersons who are emotionally exhausted due to job insecurity are likely to be even more exhausted, because they operate under higher outcome-based control and lower behavior-based control systems. Based on the preceding discussion, we advance the following hypothesis:

**Hypothesis** **2.** There is a three-way interaction between salespersons’ job insecurity perception, outcome-based control, and behavior-based control, which is mediated by emotional exhaustion, such that the positive effect of salespersons’ job insecurity perception on emotional exhaustion is strongest when outcome-based control is high and behavior-based control is low.

Our study suggests a three-way interaction mediation model to comprehensively account for the relationship between salespersons’ job insecurity and sleep quality (i.e., insomnia symptoms), such that the support provided by control systems is likely to strengthen or mitigate the indirect relationship between job insecurity and insomnia symptoms through emotional exhaustion. In our article, job demands, such as performance pressure generated from outcome-based control, mitigate the positive relationship between job insecurity and insomnia symptoms via emotional exhaustion, whereas job resources (e.g., feedback for improving salespersons’ selling skills) generated by behavior-based control reduce the positive link between job insecurity and insomnia symptoms via emotional exhaustion. Drawing on JD-R theory, we further propose that both outcome-based and behavior-based control systems play a moderating role in the mediating effect of emotional exhaustion on the relationship between job insecurity and insomnia symptoms. By adding resources and reducing demands, an outcome-based control system tends to attenuate the indirect effect of job insecurity on insomnia symptoms through emotional exhaustion. By contrast, a behavior-based control system is likely to increase job resources in handling a potential job loss situation. Under higher outcome-based and lower behavior-based control systems, job insecurity should be related to increased emotional exhaustion, leading to an increase in negative sleep quality (i.e., insomnia symptoms). Based on the preceding discussion, we advance the following hypotheses:

**Hypothesis** **3.** There is a three-way interaction between salespersons’ job insecurity, outcome-based control, and behavior-based control on insomnia symptoms, which is mediated by emotional exhaustion, such that the positive indirect effect of salespersons’ job insecurity on insomnia symptoms mediated by emotional exhaustion is strongest when outcome-based control is high and behavior-based control is low.

## 3. Methods

### 3.1. Data Collection and Sample

Our sample comprised salespersons in several sales organizations (e.g., department stores, the insurance industry, and the retail sector), who were recruited by a web-survey agency, located in the Republic of Korea. This web-survey organization possessed a subject pool of 1.2 million Republic of Koreans, who provided their occupation at the time of registration for online membership. Such web- survey panels have been employed as a credible source for accessing various samples (e.g., [43,44]). The web-survey agency allowed us to contact its members who worked in the service sector and solicit their participation in research on work environment and job stress (i.e., job insecurity and insomnia symptoms) [45]. The participants were assured of anonymity and confidentiality. We gathered their demographic information from the online survey. The participants received about USD 3 as a reward for their survey response.

We used a two-wave study model in order to reduce CMV and define the casual linkage between job insecurity and insomnia symptoms. We distributed the Time 1 (T1) survey in July 2019 and the Time 2 (T2) survey in October 2019. The T1 survey comprised items evaluating job insecurity, emotional exhaustion, output-based control, and behavior-based control, as well as positive (PA) and negative affect (NA) and social desirability biases, while the second (T2) survey included the measurement of insomnia symptoms. Of the 318 salespersons who responded to the T1 survey, 187 completed the T2 survey (retention rate = 58.8%). To test research hypotheses with a power of 0.80 and a significance level (alpha) of 0.05 [46], we calculated the optimal sample size using the G * Power sample size calculator [47], which recommended a sample size ranging between 118 and 143 for a medium effect size (0.15). Our sample size (*n* = 187) exceeded this criterion. Fifty-nine percent of the subjects were women. The average age and organizational tenure of the respondents were 37.96 (SD = 8.90) years and 5.16 years (SD = 4.65), respectively. The education level of the respondents varied: graduate school (3.7%), four-year university (42.2%), two-year college education (21.4%), and a high school education (32.6%). The subjects reported 5.16 (SD = 4.65) years of average work experience.

### 3.2. Measurement

As the original survey instruments were written in English, they were translated into Korean and then back-translated, checked, and validated by bilingual management scholars [48]. Except for insomnia symptoms, all the other variables were assessed on a five-point Likert-type scale (1 = ‘‘strongly disagree,’’ 5 = ‘‘strongly agree’’) (see Table 1).

Job insecurity. Job insecurity was measured with four items from De Witte’s [49] and Schreurs et al.’s [50] studies. An example of the items is “There is a risk that I will lose my present job in the near future.” (*α* = 0.89).

Emotional exhaustion. Emotional exhaustion was measured using four items from Maslach and Jackson’s [51] study. A sample measurement is “I feel emotionally drained from my work” (*α* = 0.83).

Insomnia symptoms. Participants responded to four items on a scale, ranging from 1 (less than once per month) to 5 (every day) [34], regarding the extent to which they experienced insomnia symptoms (i.e., sleep quality) over the previous six months [52]. An example item is “woke up several times during the night” (*α* = 0.87).

Organizational control system. Outcome-based control (e.g., “My supervisor monitors the extent to which I attain my performance goals”; α = 0.93) and behavior-based control (e.g., “My supervisor evaluates whether I provide a courteous service to customers”; α = 0.96) were each measured using three items from the study of Rodrigues et al. [37].

We employed gender, age, job tenure, education, job status, and PANAS (positive emotion (PA) and negative emotion (NA)) as control variables, due to their hidden confounding effects on emotional exhaustion (e.g., [8,53,54]) and insomnia symptoms (e.g., [55,56,57]). In addition, because our surveys treated sensitive issues (e.g., job insecurity), we needed to add social desirability bias as a covariate [8,58]. Social desirability bias was measured with five items from the short social desirability bias scale of Hays et al. [59]. Example scales include “I am always courteous, even to people who are disagreeable” and “There have been occasions when I took advantage of someone” (*α* = 0.74). Lastly, we measured PA (*α* = 0.81) and NA (*α* = 0.77) using each of the three items from “The International Positive Affect and Negative Affect Schedule Short Form” [60].

## 4. Results

### 4.1. Descrptive Stastistics, Reliability amd Validity

Table 2 provides the means, standard deviations, Cronbach’s alpha, and correlations of the research variables. The Cronbach’s alphas for the variables ranged from 0.71 to 0.90, which presents a satisfactory level of reliability [61]. To assess the convergent and discriminant validity, we used a confirmatory factor analysis (CFA) with the M-plus 8.4 software. As reported in Table 1, the suggested eight-factor model (i.e., job insecurity, outcome- and behavior-based controls, emotional exhaustion, insomnia symptoms, social desirability bias, PA, and NA) exhibited an acceptable fit in an absolute sense (χ^2^_(322)_ = 508.96; *p <* 0.05; CFI [comparative fit index] = 0.93; TLI [Tucker–Lewis index] = 0.92; RMSEA [root mean square error of approximation] = 0.06; SRMR [standardized root mean square residual] = 0.06). Furthermore, the eight constructs displayed a sufficient level of composite reliability, ranging from 0.77 to 0.91 (see Table 2). Additionally, we assessed the discriminant validity among the constructs based on Fornell and Larcker’s [62] procedure. Table 2 shows that all average variances extracted (AVEs) were larger than the squared correlation between the target construct and any of the other constructs.

As we depended on self-reported surveys, we investigated the possibility that the subjects’ responses were influenced by common method variance (CMV). CMV is defined as variance that is attributable to the systematic measurement error rather than the study constructs that the measures represent [63]. It is widely assumed that common method bias inflates relationships between variables measured by self-reports [64]. To reduce CMV, we directly assessed the social desirability bias and employed the control variable to reduce the common method bias. Furthermore, we employed Harman’s single-factor model as a statistical remedy [65]. All measures of the goodness of fit presented a worse fit for the single-factor model than for the CFA model (χ^2^_(350)_ = 1770.76; *p <* 0.05, CFI = 0.32, TLI = 0.27, RMSEA = 0.15, SRMR = 0.13). These results confirm that CMV does not pose a serious threat to the empirical rigor of our analyses.

### 4.2. Test of Hypotheses

To test our research hypotheses, we used the following steps. First, we investigated a simple mediation effect to test Hypothesis 1. Next, to estimate the moderation and moderated mediation effects (Hypothesis 2 and 3), we conducted a three-way moderated mediation analysis. Prior to the moderation and moderated analyses, all non-discrete variables were mean-centered [66]. To analyze the mediation, three-way moderation, and three-way moderated mediation effects, we used an M-plus macro developed by Hayes [67] and Stride et al. [68].

Hypothesis 1 suggested that the positive association between salespersons’ job insecurity and sleep quality (i.e., insomnia symptoms) was mediated by emotional exhaustion. We investigated the mediation hypothesis using a bootstrapping (*N* = 5000) technique, a statistical resampling procedure that estimates the standard deviation of a model from a sample [67]. The results confirmed that, controlling for several covariates, the positive effect of job insecurity on insomnia symptoms was mediated by emotional exhaustion and was significant (*b* = 0.052, 95% CI = [0.005, 0.115]). Moreover, when emotional exhaustion was included in the model, the direct effect of job insecurity on insomnia symptoms was no longer statistically significant (*b* = 0.076, 95% CI = [−0.054, 0.210]), suggesting a full mediation (see Table 3). Therefore, Hypothesis 1 was supported.

**Figure 2:**
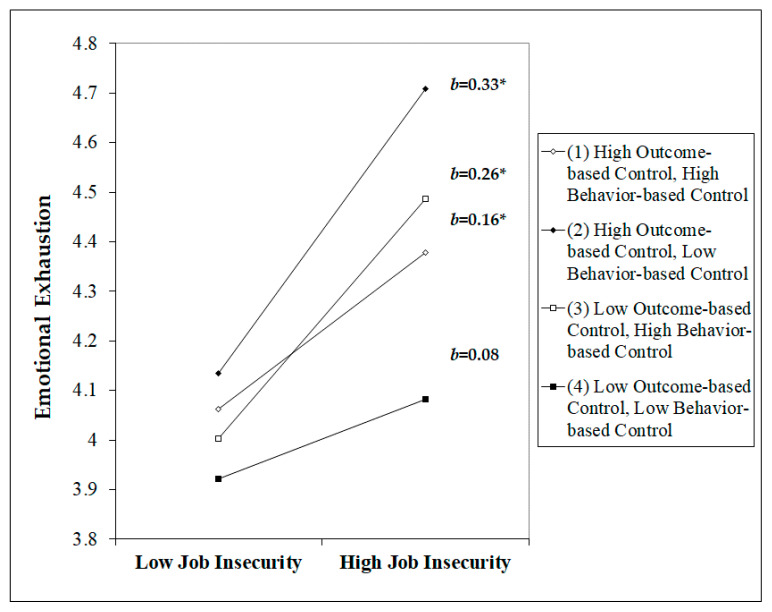
Interaction effect of outcome-based control and behavior-based control on the relationship between job insecurity and emotional exhaustion. Note: * *p* < 0.05. *b* = unstandardized coefficient.

Hypothesis 2 proposes a three-way interaction between job insecurity, outcome-based control, and behavior-based control mediated by emotional exhaustion, such that the positive relationship between job insecurity and emotional exhaustion associated with work is strongest when outcome-based control is high and behavior-based control is low. In support of Hypothesis 2, the three-way interaction between job insecurity, outcome-based control, and behavior-based control mediated by emotional exhaustion was significant (*b* = −0.08, *p <* 0.05) (see Table 4). We then conducted a simple slope analysis (plotting simple slopes at ± 1 SD of the moderator), and the three-way interaction is shown in Figure 2.

Hypothesis 3 predicts a three-way interaction between job insecurity, outcome-based control, and behavior-based control affecting insomnia symptoms through emotional exhaustion, such that the positive indirect relationship between job insecurity and sleep quality mediated by emotional exhaustion is strongest when outcome-based control is high and behavior-based control is low. This three-way-moderated mediation effect was significant (*b* = −0.038, 95% CI = [−0.083, −0.002]) (see Table 4). Furthermore, as indicated in Table 5, the indirect effect of job insecurity on insomnia symptoms through emotional exhaustion is stronger and significant (*b* = 0.136, 95% CI = [0.018, 0.323]), where there is a high level of outcome-based control and a low level of behavior-based control than under other conditions. These findings lend support to Hypothesis 3.

## 5. Discussion

We conducted this study to verify the positive mediation of emotional exhaustion between salespersons’ job insecurity and insomnia symptoms. Further, the study tested the three-way moderating effects of job insecurity, outcome-based control, and behavior-based control on the job security–emotional exhaustion link. Results showed that salespersons’ job insecurity significantly affected insomnia symptoms due to the three-month emotional exhaustion process. Furthermore, the interaction between outcome-based control and behavior-based control increased the positive relationship between job insecurity and insomnia symptoms (i.e., sleep quality) and also amplified the positive indirect effect of job insecurity on insomnia symptoms through emotional exhaustion. The positive indirect effect of job insecurity on insomnia symptoms through emotional exhaustion was stronger for a high level of outcome-based control and a low level of behavior-based control than under other conditions.

Previous research on job insecurity has proved that job insecurity leads to sleep problems [26,27], which is consistent with the findings of our study. Our study further advances the job insecurity literature by demonstrating emotional exhaustion as a mediating process between job insecurity and insomnia symptoms. Job insecurity followed by emotional exhaustion can be largely damaging to salespeople’s sleep quality since job insecurity is a major negative event at work that triggers sleep problems. As in the case of salespeople in our study, their exposure to threats of job security has often been associated with emotional exhaustion, which may heighten the frequency and the extent to which they experience sleep problems such as insomnia symptoms [27]. In addition, the three-way interaction design is one of the strengths of our study, which demonstrates that the positive effect of salespersons’ job insecurity on emotional exhaustion is strongest when outcome-based control is high and behavior-based control is low. The findings imply that such work contexts characterized by a combination of a lack of supervisor support and high levels of job pressure (e.g., high level of outcome-based control) and the substantial provision of supervisor support and low level of job pressure (e.g., behavior-based control) may attenuate the impact of job insecurity on emotional exhaustion.

### 5.1. Theoretical Implications

There has been various prior research on the effects of job insecurity, but researchers have paid relatively little attention to the role of job insecurity in sales contexts or boundary conditions. However, these factors should be considered important in that they increase or decrease the negative effect of job insecurity. In this study, we employed emotional exhaustion as a mechanism that may explain the relationship between salespersons’ job insecurity and impaired sleep. This study demonstrated that the effect of job insecurity is strong enough to have a long-term negative impact on the quality of sleep for salespersons. Furthermore, we sought to develop a model that addresses the effect of job insecurity and its boundary conditions in the sales domain. The strength of our research design is that we organized the research sample to include participants from various sales organizations. This research design increases the generalizability of our findings to the entire sales sector.

Above all, our study revealed how the quality of sleep of service employees experiencing job insecurity decreased, and under what conditions these negative effects were exacerbated. These findings provide important insights into how to manage salespersons’ job insecurity. Another contribution of our research is that we have enhanced our theoretical understanding of job insecurity by adopting the AET and JD-R theories as important theoretical frameworks. Consistent with AET’s explanation, our findings confirmed that negative emotional responses (i.e., emotional fatigue) serve as central mediators of job insecurity’s positive effects on insomnia symptoms. Besides, our research verified that organizational control systems exert a mediation effect. This finding supports the JD-R proposition that work context facilitates the effect of workplace events on affective response. The results demonstrate that the negative impact of workplace events (e.g., job insecurity) can be increased by a higher job demand (e.g., outcome-based control) and a lower job resource (e.g., behavior-based control). Our findings support AET in that they suggest that job insecurity is a critical work stressor that depletes the emotional resources for sleep quality. Consistent with AET, results showed that salespersons who are depleted of resources due to job insecurity are more sensitive to loss of social resources and more emotionally exhausted. Moreover, we confirmed that salespersons’ sleep quality decreased when they were exposed to job demand. Accordingly, this study expands the current theoretical explanation of job security by introducing AET and JD-R as a framework for addressing servicepersons’ job insecurity.

Our study also suggests a comprehensive explanation of the roles of organizational control systems in job insecurity through job demands and resources, as the boundary conditions of job insecurity. This research first investigates a combination effect of outcome-based and behavior-based controls on the relationship between job insecurity and employee outcomes. Job insecurity research adopting the JD-R framework has identified a combination of outcome-based and behavior-based controls moderating the positive impact of job insecurity on insomnia symptoms. When outcome-based control and behavior-based control were combined, the effects of job insecurity perception on sleep disorders mediated by emotional exhaustion were changed. These results strongly prove that the effects of job insecurity become worse in situations where salespersons get higher outcome-based control and lower behavior-based control. In addition, the findings suggest that providing adequate behavior-based controls and also preventing or reducing outcome-based controls helps employees cope with job insecurity.

### 5.2. Practical Implications

Research findings offer some practical implications for job insecurity management in the sales sector. As our research reveals, job insecurity is a relevant work stressor that causes salespersons’ emotional exhaustion and impaired sleep quality. Freeing salespersons from concerns about job insecurity is likely to be the best solution for job insecurity issues. However, digital technologies are rapidly replacing salespersons’ roles in recent years, which makes job insecurity an unavoidable phenomenon for salespersons [2,8]. In order to minimize salespersons’ perceptions of job insecurity, sales organizations should offer appropriate interventions or training programs to enhance employees’ confidence and self-efficacy [1]. Furthermore, to reduce emotional exhaustion due to job insecurity, sales organizations also might consider offering a diverse range of personal-level resources (e.g., develop programs to enhance job calling, emotional resilience, and control quotient) and organizational-level resources (e.g., prepare formal rules and informal norms to promote empowerment).

Second, sales managers must have a clear understanding of the conditions which can exacerbate or elevate the effects of job insecurity on employee outcomes. In this study, organizational control systems (i.e., outcome-based and behavior-based controls) turned out to present such boundary conditions. While the majority of sales organizations implement a mixed approach of combining both outcome-based and behavior-based controls to accomplish desirable goals (e.g., [20,23]), previous studies have not provided substantial guidelines for an effective design and implementation [19]. Our results demonstrated that a trade-off moderating the effects of outcome-based and behavior-based controls exists between job insecurity perception and affective response. While previous researchers have shown that output control might trigger short-term thinking [19], competitive pressure or a short-term sales focus may also engender negative psychological and psychical responses (e.g., emotional exhaustion, ego-depletion, and sleep difficulties), which can potentially damage job-related confidence or self-efficacy. However, behavioral control requires managers to be actively involved in directing, training, and evaluating their employees. [20]. When sales organizations train and direct their salespersons in a situation of high job insecurity, salespersons may present signals, such as long-term employment. Thus, sales organizations must design appropriate control mechanisms when managing salespersons.

## 6. Limitations and Directions for Future Studies

The limitations of this study are as follows. First, it should be noted that while there are many factors associated with sleep quality (e.g., insomnia symptoms), we only examined sleep in a general sense. As sleep is conceptualized in terms of quantity [28] and quality [17,30], it is necessary to disentangle the different relationships between job insecurity, emotional exhaustion, sleep quality, and sleep quantity.

Second, although we revealed emotional exhaustion as a bridging process between job insecurity and sleep quality, we did not employ affective or cognitive responses as mediators. As suggested in the study by Bernhard-Oettel et al. [26], procedural justice mediates the positive relationship between job insecurity and sleep difficulties in the within-person model. Therefore, to elaborate the relationship between job insecurity and sleep problems, we recommend introducing several mediators and testing them as a future study agenda.

Third, there may exist other mediators (e.g., work engagement, ego depletion, job stress, and burnout) between job insecurity and insomnia symptoms. Besides, potential moderators (e.g., emotion regulation ability, emotional intelligence, and mindfulness) can increase or decrease the strength of the relationship between job insecurity and emotional exhaustion.

Fourth, as we only collected data from service employees, our sample may not represent the entire employee population. While job stress research has stressed the importance of job insecurity in the service sector (e.g., [69]), the relationship between job insecurity, emotional exhaustion, sleep, and organizational control systems needs to be validated in more diverse industries. In addition, because frontline service employees’ jobs are resource-draining, they require more job resources and fewer job demands. For this reason, the effect of organizational control systems may have turned out to be significant in our sample. However, to accurately capture the effect of outcome-based and behavior-based controls on job insecurity and sleep quality mediated by emotional exhaustion, more diverse samples from different occupations need to be considered.

Fifth, future research may consider the side-effects of behavior-based control on emotional exhaustion and sleep quality. Some studies have also suggested that tight control and close attention of supervisors under a behavior-based control system make employees have less freedom and empowerment for strategy adoption in customer interactions and service provision [18,36]. Tight direction and emotionally demanding interactions with supervisors could be another source of job demand that boosts salespeople’s emotional exhaustion in the context of job incivility. Future studies must consider these side-effects of behavior-based control on employee outcomes.

Finally, the severity of job insecurity in South Korea [70] may have contributed to our findings of the strong negative effect in the sample of South Korean employees. Overall, we recommend that future scholars cross-validate our findings with diverse samples from different countries and regions. Furthermore, as the sample size of this study was small, all results of this research should be interpreted cautiously. Further studies must attempt to replicate these findings using a large sample.

## 7. Conclusions

This study contributes to the advancement of job insecurity research by developing and testing a moderated mediation model of job insecurity using South Korean service employee samples. Our findings add meaningful value to the theoretical advances in related research by delineating boundary conditions that strengthen or weaken the relationship between job insecurity and insomnia symptoms through emotional exhaustion. Although we focused on outcome-based control and behavior-based control as a key source of organizational control, by conducting future investigations that introduce other forms of organizational control (e.g., ability control or normative control) as additional moderators, researchers can supplement the results of the current research and enrich the understanding of boundary conditions affecting job insecurity.

## Figures and Tables

**Table 1:** The measurement scales results of confirmatory factor analysis.

Construct	Measurement Items	λ
Job Insecurity	I am sure that I will be able to keep my job. ^®^	0.85
	There is a risk that I will lose my present job in the near future.	0.83
	I feel uncertain about the future of my job.	0.73
	I think that I will lose my job in the near future.	0.90
Emotional Exhaustion	I feel emotionally drained from my work.	0.83
	I feel fatigued when I get up in the morning and have to face another day on the job.	0.75
	I feel burned out from my work.	0.76
Insomnia Symptoms	I had trouble falling asleep.	0.82
	I had trouble staying asleep (including waking up too early).	0.82
	I woke up several times during the night.	0.92
	I woke up after my usual amount of sleep feeling tired and worn out.	0.61
Outcome-based Control	My supervisor establishes specific performance goals for my job.	0.75
	My supervisor monitors the extent to which I attain my performance goals.	0.84
	If my performance goals were not met, I would be required to explain why.	0.66
Behavior-based Control	My supervisor evaluates whether I provide a courteous service to customers.	0.86
	My supervisor evaluates my ability to deal innovatively with unique situations and/or discover customer needs.	0.93
	My commitment to customers is evaluated by my supervisor.	0.83
Positive Affectivity	Active	0.74
	Attentive	0.89
	Alert	0.69
Negative Affectivity	Nervous	0.79
	Scared	0.88
	Ashamed	0.55
Social Desirability Bias	I am always courteous, even to people who are disagreeable.	0.63
	There have been occasions when I took advantage of someone.	0.50
	I sometimes try to get even, rather than forgive and forget.	0.62
	I sometimes feel resentful when I don’t get my way.	0.69
	No matter whom I’m talking to, I’m always a good listener.	0.69
χ^2^_(322)_ = 508.96; *p <* 0.05, CFI = 0.93, TLI = 0.92, RMSEA = 0.06, SRMR = 0.06		

Note: ® = reversed scale.

**Table 2:** Means, standard deviations, and correlations.

	M	SD	α	CR	1	2	3	4	5	6	7	8	9	10	11	12	13
1. Gender	0.41	0.49	−	−	−												
2. Age	37.96	8.90	−	−	0.07	−											
3. Education (year)	14.34	1.87	−	−	0.16 *	0.04	−										
4. Job Tenure	5.16	4.65	−	−	0.05	0.42 **	0.05	−									
5. Job Status	0.65	0.48	−	−	−0.13 ^†^	−0.17 *	0.05	−0.06	−								
6. Social Desirability Bias	3.56	0.55	0.71	0.77	0.01	−0.03	0.09	−0.11	0.05	0.40							
7. Positive Affectivity	2.51	0.84	0.81	0.82	0.18 *	0.02	0.08	−0.12	0.08	0.20 **	0.61						
8. Negative Affectivity	2.90	0.90	0.77	0.79	−0.16 *	−0.33 **	−0.05	−0.08	0.02	−0.05	−0.15 *	0.57					
9. Job Insecurity	2.80	0.95	0.89	0.90	0.02	−0.00	−0.10	0.06	−0.11	−0.04	−0.17 *	0.18 *	0.69				
10. Emotional Exhaustion	3.28	0.81	0.83	0.82	−0.21 **	−0.31 **	−0.21 **	−0.06	0.06	−0.08	−0.33 **	0.56 **	0.26 **	0.61			
11. Insomnia Symptoms	2.93	0.92	0.87	0.88	−0.17 *	0.02	−0.16 *	0.03	−0.08	−0.24 **	−0.13 ^†^	0.20 **	0.18 *	0.38 **	0.64		
12. Outcome-Based Control	2.91	0.93	0.79	0.80	0.10	0.06	0.01	0.03	−0.05	0.20 **	0.29 **	0.02	−0.06	0.03	−0.01	0.57	
13. Behavior-Based Control	3.01	0.96	0.90	0.91	0.10	−0.05	0.05	0.01	0.03	0.18 *	0.23 **	0.07	−0.01	0.07	−0.03	0.55 **	0.76

Note: ^†^ *p <* 0.10, * p < 0.05, ** p < 0.01. Numbers along the diagonal are the average variances extracted (AVEs). CR = composite reliability. Gender: 0 = female, 1 = male. Employment status: 0 = contract, 1 = permanent.

**Table 3:** Test of the mediation effect of emotional exhaustion.

Path	Effect *(b)*	95% CI_low_	95% CI_high_
Total Effect			
Job Insecurity → Insomnia Symptoms	0.128	−0.019	0.273
Direct Effect			
Job Insecurity → Insomnia Symptoms	0.076	−0.054	0.210
Indirect Effect			
Job Insecurity → Emotional Exhaustion → Insomnia Symptoms	0.052	0.005	0.115

Note: the coefficient is unstandardized.

**Table 4:** Test of the moderating effects of outcome-based and behavior-based controls.

Variables	Emotional Exhaustion		Insomnia Symptoms	
	b(se)		b(se)	
Gender	−0.09	(0.09)	−0.22	(0.13) ^†^
Age	−0.02	(0.01) *	0.01	(0.01) ^†^
Education	−0.06	(0.02) **	−0.02	(0.03)
Job Tenure	0.00	(0.01)	−0.01	(0.01)
Job Status	0.16	(0.09) ^†^	−0.17	(0.13)
Social Desirability Bias	0.06	(0.09)	−0.34	(0.11) **
Positive Affectivity	−0.26	(0.06) **	0.09	(0.08)
Negative Affectivity	0.36	(0.05) **	−0.01	(0.08)
Job Insecurity	0.20	(0.06) **	0.08	(0.07)
Outcome-Based Control (OC)	0.11	(0.06) ^†^		
Behavior-Based Control (BC)	0.01	(0.06)		
Job Insecurity × OC	0.04	(0.06)		
Job Insecurity × BC	0.01	(0.06)		
OC × BC	−0.12	(0.05) **		
Job Insecurity × OC × BC	−0.08	(0.04) *		
Emotional Exhaustion			0.45	(0.10) **
*R* ^2^	49.3%		23.9%	
Moderated Mediation Index				
Job Insecurity × OC × BC → Emotional Exhaustion → Insomnia Symptoms: *b* = −0.038, 95% CI = [−0.083, −0.002]				

Note: ^†^ *p* < 0.10, * *p* < 0.05, ** *p* < 0.01. The coefficient is unstandardized.

**Table 5:** Test of the conditional indirect effect of job insecurity on insomnia symptoms through emotional exhaustion with different levels of outcome-based control and behavior-based control.

Moderating Variables				
Outcome-Based Control	Behavior-Based Control	*b*	Cl_95%low_	Cl_95%high_
Low Level	Low Level	0.038	−0.028	0.133
Low Level	High Level	0.113	0.013	0.256
High Level	Low Level	0.136	0.018	0.323
High Level	High Level	0.076	0.019	0.162

Note. CI = confidence interval; *b* = unstandardized coefficient. High = M + 1 SD, Low = M−1 SD.

## References

[1] Lam C.F., Liang J., Ashford S.J., Lee C. (2015). Job insecurity and organizational citizenship behavior: Exploring curvilinear and moderated relationships. J. Appl. Psychol..

[2] Wang H.J., Lu C.Q., Siu O.L. (2015). Job insecurity and job performance: The moderating role of organizational justice and the mediating role of work engagement. J. Appl. Psychol..

[3] Chaker N.N., Schumann D.W., Zablah A.R., Flint D.J. (2016). Exploring the state of salesperson insecurity: How it emerges and why it matters?. J. Mark. Theory Pract..

[4] Bouzari M., Karatepe O.M. (2018). Antecedents and outcomes of job insecurity among salespersons. Mark. Intell. Plan..

[5] Cheng G.H., Chan D.K. (2008). Who suffers more from job insecurity? A meta-analytic review. Appl. Psychol..

[6] Shoss M.K. (2017). Job insecurity: An integrative review and agenda for future research. J. Manag..

[7] Darvishmotevali M., Arasli H., Kilic H. (2017). Effect of job insecurity on frontline employee’s performance. Int. J. Contemp. Hosp. Manag..

[8] Shin Y., Hur W.M. (2019). When do service employees suffer more from job insecurity? The moderating role of coworker and customer incivility. Int. J. Environ. Res. Public Health.

[9] Zeytinoglu I.U., Keser A., Yılmaz G., Inelmen K., Özsoy A., Uygur D. (2012). Security in a sea of insecurity: Job security and intention to stay among service sector employees in Turkey. Int. J. Hum. Resour. Manag..

[10] Delpechitre D., Beeler L. (2018). Faking it: Salesperson emotional intelligence’s influence on emotional labor strategies and customer outcomes. J. Bus. Ind. Mark..

[11] Kwak H., Anderson R.E., Leigh T.W., Bonifield S.D. (2019). Impact of salesperson macro-adaptive selling strategy on job performance and satisfaction. J. Bus. Res..

[12] Rapp A., Ahearne M., Mathieu J., Schillewaert N. (2006). The impact of knowledge and empowerment on working smart and working hard: The moderating role of experience. Int. J. Res. Mark..

[13] Ktenas N. (2014). Employment and Labour Law.

[14] Díaz E., Martín-Consuegra D., Esteban Á. (2017). Sales agents vs. the internet: Understanding service sabotage based on the conservation of resources theory. Internet Res..

[15] Sharma D., Gassenheimer J.B. (2009). Internet channel and perceived cannibalization: Scale development and validation in a personal selling context. Eur. J. Mark..

[16] Weiss H.M., Cropanzano R., Staw B.M., Cummings L.L. (1996). Affective events theory: A theoretical discussion of the structure, causes, and consequences of affective experiences at work. Research in Organizational Behavior.

[17] Litwiller B., Snyder L.A., Taylor W.D., Steele L.M. (2017). The relationship between sleep and work: A meta-analysis. J. Appl. Psychol..

[18] Crosno J.L., Brown J.R. (2015). A meta-analytic review of the effects of organizational control in marketing exchange relationships. J. Acad. Mark. Sci..

[19] Wang G., Dou W., Zhou N. (2012). The interactive effects of sales force controls on salespersons behaviors and customer outcomes. J. Pers. Sell. Sales Manag..

[20] Miao C.F., Evans K.R. (2014). Motivating industrial salesforce with sales control systems: An interactive perspective. J. Bus. Res..

[21] Cravens D.W., Lassk F.G., Low G.S., Marshall G.W., Moncrief W.C. (2004). Formal and informal management control combinations in sales organizations: The impact on salesperson consequences. J. Bus. Res..

[22] Jaworski B.J., Stathakopoulos V., Krishnan H.S. (1993). Control combinations in marketing: Conceptual framework and empirical evidence. J. Mark..

[23] Onyemah V., Anderson E. (2009). Inconsistencies among the constitutive elements of a sales force control system: Test of a configuration theory–based performance prediction. J. Pers. Sell. Sales Manag..

[24] Schaufeli W.B., Bakker A.B. (2004). Job demands, job resources, and their relationship with burnout and engagement: A multi-sample study. J. Organ. Behav. Int. J. Ind. Occup. Organ. Psychol. Behav..

[25] De Cuyper N., De Witte H., Vander Elst T., Handaja Y. (2010). Objective threat of unemployment and situational uncertainty during a restructuring: Associations with perceived job insecurity and strain. J. Bus. Psychol..

[26] Bernhard-Oettel C., Eib C., Griep Y., Leineweber C. (2019). How do job insecurity and organizational justice relate to depressive symptoms and sleep difficulties: A multilevel study on immediate and prolonged effects in Swedish workers. Appl. Psychol..

[27] Kim Y.K., Kramer A., Pak S. (2020). Job insecurity and subjective sleep quality: The role of spillover and gender. Stress Health.

[28] Barnes C.M. (2012). Working in our sleep: Sleep and self-regulation in organizations. Organ. Psychol. Rev..

[29] Wickens C.D., Hutchins S.D., Laux L., Sebok A. (2015). The impact of sleep disruption on complex cognitive tasks: A meta-analysis. Hum. Factors.

[30] Cappuccio F.P., D’Elia L., Strazzullo P., Miller M.A. (2010). Quantity and quality of sleep and incidence of type 2 diabetes: A systematic review and meta-analysis. Diabetes Care.

[31] Harvey A.G., Stinson K., Whitaker K.L., Moskovitz D., Virk H. (2008). The subjective meaning of sleep quality: A comparison of individuals with and without insomnia. Sleep.

[32] Pilcher J.J., Ginter D.R., Sadowsky B. (1997). Sleep quality versus sleep quantity: Relationships between sleep and measures of health, well-being and sleepiness in college students. J. Psychosom. Res..

[33] Schmitt A., Belschak F.D., Den Hartog D.N. (2017). Feeling vital after a good night’s sleep: The interplay of energetic resources and self-efficacy for daily proactivity. J. Occup. Health Psychol..

[34] Jenkins C.D., Stanton B.A., Niemcryk S.J., Rose R.M. (1988). A scale for the estimation of sleep problems in clinical research. J. Clin. Epidemiol..

[35] Selenko E., Mäkikangas A., Mauno S., Kinnunen U. (2013). How does job insecurity relate to self-reported job performance? Analysing curvilinear associations in a longitudinal sample. J. Occup. Organ. Psychol..

[36] Evans K.R., Landry T.D., Li P.C., Zou S. (2007). How sales controls affect job-related outcomes: The role of organizational sales-related psychological climate perceptions. J. Acad. Mark. Sci..

[37] Rodrigues LC A., Coelho F.J., Sousa C.M. (2015). Control mechanisms and goal orientations: Evidence from frontline service employees. Eur. J. Mark..

[38] Santini F., Vieira V.A., Ladeira W.J., Sampaio C.H. (2019). Behaviour-Based and Outcome-Based Control Systems: A Meta-Analytic Study. Can. J. Adm. Sci. Rev. Can. Sci. Adm..

[39] Agarwal S., Ramaswami S.N. (1993). Marketing controls and employee responses: The moderating role of task characteristics. J. Acad. Mark. Sci..

[40] Oliver R.L., Anderson E. (1994). An empirical test of the consequences of behavior−and outcome-based sales control systems. J. Mark..

[41] Li M., Peng L., Zhuang G. (2020). Sales control systems and salesperson commitment: The moderating role of behavior uncertainty. Sustainability.

[42] Choi N.H., Dixon A.L., Jung J.M. (2005). Dysfunctional behavior among sales representatives: The effect of supervisory trust, participation, and information controls. J. Pers. Sell. Sales Manag..

[43] Landers R.N., Behrend T.S. (2015). An inconvenient truth: Arbitrary distinctions between organizational, Mechanical Turk, and other convenience samples. Ind. Organ. Psychol..

[44] Roulin N. (2015). Don’t throw the baby out with the bathwater: Comparing data quality of crowdsourcing, online panels, and student samples. Ind. Organ. Psychol..

[45] Ford M.T., Jin J. (2015). Incongruence between workload and occupational norms for time pressure predicts depressive symptoms. Eur. J. Work Organ. Psychol..

[46] Cohen J. (1988). Statistical Power Analysis for The Behavioral Sciences.

[47] Faul F., Erdfelder E., Lang A.G., Buchner A. (2007). G* Power 3: A flexible statistical power analysis program for the social, behavioral, and biomedical sciences. Behav. Res. Methods.

[48] Brislin R.W. (1970). Back-translation for cross-cultural research. J. Cross Cult. Psychol..

[49] De Witte H., Bouwen R., DeWitte K., de Witte H., Taillie T.U. (2000). Arbeidsethos en jobonzekerheid: Meting en gevolgen voor welzijn, tevredenheid en inzet op het werk [Work ethic and job insecurity: Measurement and consequences for wellbeing, satisfaction and performance]. Van Groep Naar Gemeenschap [From Group to Community]: Liber Amicorum prof. Dr. Leo Lagrou.

[50] Schreurs B., Van Emmerik H., Notelaers G., De Witte H. (2010). Job insecurity and employee health: The buffering potential of job control and job self-efficacy. Work Stress.

[51] Maslach C., Jackson S.E. (1981). The measurement of experienced burnout. J. Occup. Behav..

[52] Demsky C.A., Fritz C., Hammer L.B., Black A.E. (2019). Workplace incivility and employee sleep: The role of rumination and recovery experiences. J. Occup. Health Psychol..

[53] Rhee S.Y., Hur W.M., Kim M. (2017). The relationship of coworker incivility to job performance and the moderating role of self-efficacy and compassion at work: The Job Demands-Resources (JD-R) Approach. J. Bus. Psychol..

[54] Moon T.W., Hur W.M., Choi Y.J. (2019). How leaders’ perceived emotional labor leads to followers’ job performance. J. Serv. Theory Pract..

[55] Fritz C., Park Y., Shepherd B.R. (2019). Workplace incivility ruins my sleep and yours: The costs of being in a work-linked relationship. Occup. Health Sci..

[56] Lee S., Mogle J.A., Jackson C.L., Buxton O.M. (2019). What’s not fair about work keeps me up: Perceived unfairness about work impairs sleep through negative work-to-family spillover. Soc. Sci. Res..

[57] Toker S., Laurence G.A., Fried Y. (2015). Fear of terror and increased job burnout over time: Examining the mediating role of insomnia and the moderating role of work support. J. Organ. Behav..

[58] Valentine S., Fleischman G. (2018). From schoolyard to workplace: The impact of bullying on sales and business employees’ machiavellianism, job satisfaction, and perceived importance of an ethical issue. Hum. Resour. Manag..

[59] Hays R.D., Hayashi T., Stewart A.L. (1989). A five-item measure of socially desirable response set. Educ. Psychol. Meas..

[60] Thompson E.R. (2007). Development and validation of an internationally reliable short-form of the positive and negative affect schedule (PANAS). J. Cross Cult. Psychol..

[61] Nunnally J.C. (1978). Psychometric Theory.

[62] Fornell C., Larcker D.F. (1981). Evaluating structural equation models with unobservable variables and measurement error. J. Mark. Res..

[63] Bagozzi R.P., Yi Y. (1990). Assessing method variance in multitrait-multimethod matrices: The case of self-reported affect and perceptions at work. J. Appl. Psychol..

[64] Conway J.M., Lance C.E. (2010). What reviewers should expect from authors regarding common method bias in organizational research. J. Bus. Psychol..

[65] Podsakoff P.M., MacKenzie S.B., Podsakoff N.P. (2012). Sources of method bias in social science research and recommendations on how to control it. Annu. Rev. Psychol..

[66] Aiken L., West S. (1991). Multiple Regression: Testing and Interpreting Interactions.

[67] Hayes A.F. (2017). Introduction to Mediation, Moderation, and Conditional Process analysis: A Regression-based Approach.

[68] Stride C.B., Gardner S., Catley N., Thomas F. Mplus Code for the Mediation, Moderation, and Moderated Mediation Model Templates from Andrew Hayes. Process Analysis Examples. https://offbeat.group.shef.ac.uk/FIO/mplusmedmod.htm.

[69] Shin Y., Hur W.M. (2020). Supervisor incivility and employee job performance: The mediating roles of job insecurity and amotivation. J. Psychol..

[70] Kim Y., Kim S.S. (2018). Job insecurity and depression among automobile sales workers: A longitudinal study in South Korea. Am. J. Ind. Med..

